# Supplementary material for: Gene Expression and Mutational Profile in BAP-1 Inactivated Melanocytic Lesions of Progressive Malignancy from a Patient with Multiple Lesions
Source: Genes (Basel). 2021 Dec 22;13(1):10. doi: 10.3390/genes13010010 (PMC8774463; doi:10.3390/genes13010010)
Supplement: Supplementary file 1 [file genes-13-00010-s001.zip › Suppl table.pdf]

Supplemental Table 1. Selected most significantly differentially expressed (upregulated) pathways and the involved genes among the lesions

Pathway	Atypical tumor vs Nevus	Melanoma vs Atypical tumor	Melanoma vs Nevus
Transmembrane receptor protein kinase acitivity	ROS1, IGF1R, NTRK3, EPHA5	ACVR1C, KIT, EPHA5	IGF1R, KIT, ALK, EPHA5
Protein kinase activity	ROS1, TNF, IGF1R, FZD5, NTRK3, WNT11, DUSP9, EPHA5	CDKN1C, DGKB, FGF13, ACVR1C, GRM1, KIT, CDK2, EPHA5	CDKN1C, IGF1R, FZD5, WNT11, DUSP9, HIPK2, KIT, ALK, CDK2, EPHA5
MAP kinase activity	TNF, IGF1R, FZD5, NTRK3, DUSP9		IGF1R, FZD5, DUSP9, KIT, ALK
Protein tyrosine kinase activity	ROS1,IGF1R,NTRK3,EPHA5		IGF1R,HIPK2,KIT,ALK,EPHA5
Sequence-specific DNA binding	HOXA11, SOX10, ETV5, IRF4,NCL,PAX3,PRDM7,TFAP2B		HOXA10,SOX10,HOXA11, MITF,ETV4,ETV5,IRF4, PAX3, PRDM7,TFAP2B
Regulatory region nucleic acid binding	SOX10,ETV5,IRF4,TNF,PRDM7,TFAP2B,WNT11		HOXA10, SOX10,WNT11,MITF,ETV4, ETV5, IRF4,PRDM7,TFAP2B
Androsterone dehydrogenase (B-specific) activity	AKR1C1		AKR1C1
Endothelin receptor activity	EDNRB		EDNRB
17-alpha,20-alpha-dihydroxypregn-4-en-3-one dehydrogenase activity	AKR1C1		AKR1C1
Molecular transducer activity		MPL,CDKN1C,CD36,ACVR1C,GRM1,LGR5,KIT,LEF1,CDK2,EPHA5	MPL,CDKN1C,KRT17,RASGRF1,TNFRSF14,EDNRB,IGF1R,FZD5,KIT,ALK,CDK2,EPHA5
Phosphotransferase activity, alcohol group as acceptor		CDKN1C,DGKB,FGF13,ACVR1C,GRM1,KIT,CDK2,EPHA5	CDKN1C,WNT11,DUSP9,HIPK2, IGF1R,PIK3CD,FZD5,KIT,ALK,CDK2,EPHA5
Thrombopoietin receptor activity		MPL	MPL
Transferase activity, transferring phosphorus-containing groups		CDKN1C,DGKB,FGF13,ACVR1C,GRM1,KIT,CDK2,EPHA5	CDKN1C,WNT11,DUSP9,HIPK2, IGF1R,PIK3CD,FZD5,KIT,ALK,CDK2,EPHA5
Signaling pathways regulating pluripotency of stem cells		IGF1R,FZD5,WNT11,DUSP9	IGF1R,PIK3CD,FZD5,WNT11,DUSP9
