# Supplementary figures and images for: Gene Expression and Mutational Profile in BAP-1 Inactivated Melanocytic Lesions of Progressive Malignancy from a Patient with Multiple Lesions
Source: Genes (Basel). 2021 Dec 22;13(1):10. doi: 10.3390/genes13010010 (PMC8774463; doi:10.3390/genes13010010)

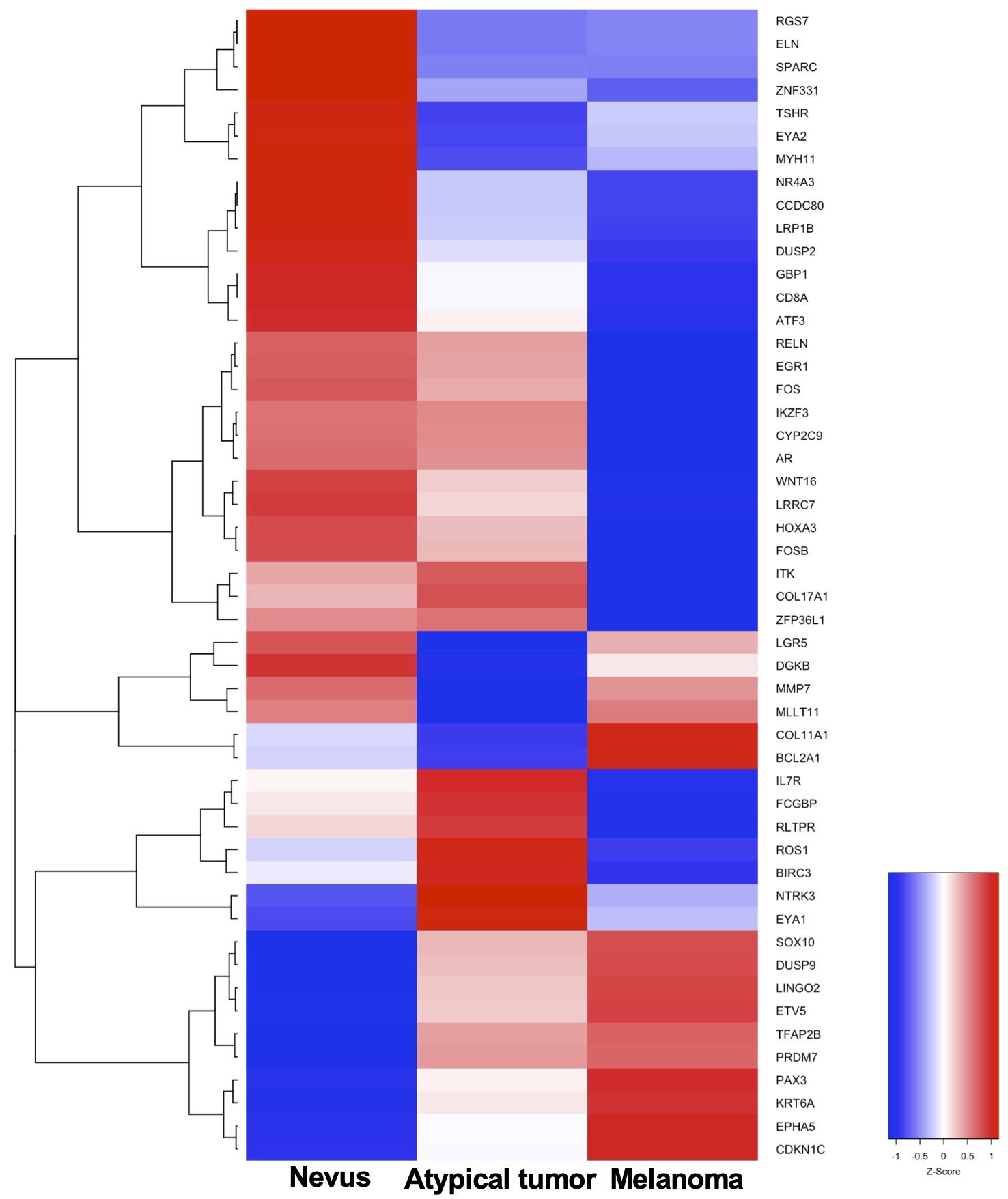

Supplement: Supplementary file 1 [file genes-13-00010-s001.zip › Suppl Figure 1.jpg]

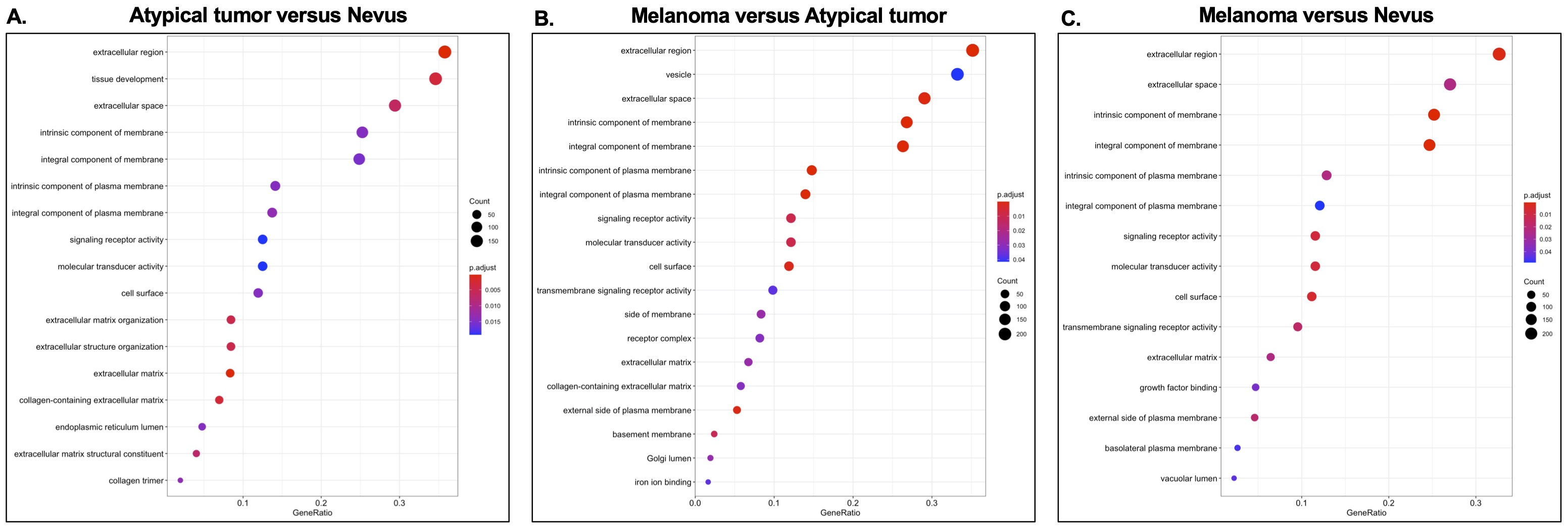

Supplement: Supplementary file 1 [file genes-13-00010-s001.zip › Suppl Figure 2.jpg]
